# Emotion Regulation and Parent Co-Regulation in Children with Autism Spectrum Disorder

**DOI:** 10.1007/s10803-016-3009-9

**Published:** 2017-01-09

**Authors:** Victoria Ting, Jonathan A. Weiss

**Affiliations:** Department of Psychology, York University, 4700 Keele Street, Toronto, ON M3J 1P3 Canada

**Keywords:** Emotion regulation, Autism, Parents, Emotional problems

## Abstract

Children with autism spectrum disorder (ASD) often exhibit emotional problems, which can be associated with emotion regulation (ER) difficulties. Parent co-regulation is often associated with child ER and emotional problems, though little work has been done with reference to youth with ASD. This study investigated the association among parent co-regulation, child ER, and internalizing and externalizing problems in 51 parents and school-aged children with ASD. Parent co-regulation strategies and scaffolding were not associated with parent-reported levels of child internalizing problems. Parent scaffolding and child ER predicted externalizing problems, after controlling for child age and IQ. Suggestions for future research on parent involvement in the emotional development of children with ASD are discussed, as well as implications for ER-focused interventions.

## Introduction

Individuals with Autism Spectrum Disorder (ASD) experience impairment in social interaction and communication (American Psychiatric Association 2013), and often have externalizing (e.g., aggression, hyperactivity) and internalizing (e.g., anxiety, depression) emotional problems. Children and adolescents with ASD have higher levels of emotional difficulties than typically developing children (Dickerson Mayes et al. 2011) and those with intellectual disability (Brereton et al. 2006), with rates often ranging between 71 and 86% (Ooi et al. 2011; Totsika et al. 2011). Over half of children with ASD have been reported to experience four or more externalizing and internalizing problems concurrently and frequently (Maskey et al. 2013). These problems can significantly impact children’s quality of life and achievement, as well as the health and wellbeing of their families (Gadow et al. 2008; Wood and Gadow 2010).

### Emotion Regulation

For some youth, the broad externalizing and internalizing difficulties outlined above may be explained by underlying deficits in emotion regulation (i.e., the set of processes that control emotions; Gross and Thompson 2007; Mazefsky et al. 2013; Rieffe et al. 2011; Weiss 2014). Children with ASD tend to use more maladaptive emotion regulation strategies (e.g., venting, avoidance) in frustrating situations than typically developing matched controls (Jahromi et al. 2012; Konstantareas and Stewart 2006). In adolescence, both typically developing youth and those with ASD report similar levels of adaptive, voluntary forms of emotion regulation (e.g., problem solving, emotional control), but those with ASD report higher levels of involuntary emotion regulation strategies that are generally considered to be maladaptive (e.g., rumination, intrusive thoughts, physiological and emotional arousal, mind going blank and numb) (Mazefsky et al. 2014). Such involuntary forms of emotion regulation are related to higher levels of internalizing and externalizing problems, as well as symptoms of anxiety and depression in children (Mazefsky et al. 2014). Emotion regulation deficits are also found in adults with ASD (Samson et al. 2012), suggesting that the difficulties with controlling emotions seen in childhood are also observed later on in life.

A recent systematic review of emotion regulation in children with ASD found that research has largely relied on self-report (38%) or informant report (44%); fewer used naturalistic observation/behavior coding (31%) or open-ended measures (13%); and only two (6%) of the studies explored correlates of emotion regulation (Weiss et al. 2014). Self-report in children with ASD may be problematic due to the lack of correspondence with parent report (Mazefsky et al. 2011; Meyer et al. 2006; White et al. 2009) and with physiological measures (Shalom et al. 2006), raising the question of the validity of self-report responses in this population. Parent report is often used instead of children’s self-report, but relying on one informant (e.g., parent report) for outcome and predictor variables can lead to overestimates of associations because of common method variance (Lindell and Whitney 2001; Richardson et al. 2009).

Parents can be important contributors to their children’s emotion regulation. Higher levels of positive parenting behaviors have been associated with lower levels of child externalizing behavior problems in typically developing children (Boeldt et al. 2012; Maljaars et al. 2014), and there is some research to indicate similar patterns among children with ASD. Internalizing and externalizing problems in children with ASD have been associated with several parental and family factors, important considerations given that individual child characteristics often account for only a small amount of variance in psychopathology (Gadow et al. 2008; Mayes et al. 2011; Sukhodolsky et al. 2008). One study of children with developmental disabilities, 2.5–5 years of age, found that parent disciplining practices and parent–child attachment were associated with improvements in child self-control (Lewallen and Neece 2015). Similarly, Boonen and colleagues (2014) found that lower levels of negative or controlling parenting (i.e., discipline and harsh punishment) was associated with fewer externalizing behavior problems in children with ASD. Fewer behavior problems in children with ASD have also been associated with the absence of a family history of psychopathology (Gadow et al. 2008), low levels of expressed emotion (i.e., high criticism and/or emotional over-involvement; Greenberg et al. 2006) and better family adaptability (i.e., the ability to respond to a stressor using strategies such as problem-solving, changing roles and responsibilities; Baker et al. 2011).

Parents also play an important role in supporting the development of emotion regulation skills. From a theoretical perspective, Sameroff and Fiese’s (2000) model describes development as a series of transactional relations between self-regulation and other-regulation over time, whereby a child progresses from relying on others to regulate their needs and emotions to being able to regulate themselves. The relationship between self- and other-regulation is “transactional” in that an individual’s ability to self-regulate is influenced by how their caregiver helped them regulate earlier in life. In a study of typically developing school-aged children, parents with high levels of unsupportive responses to emotions rated their children as having poorer emotion regulation and more depressive symptoms (Sanders et al. 2015). Many parents of children with ASD report that they notice that their own emotions have an effect on their child’s emotions and behaviors, and vice versa; a phenomenon that has been called “emotional transmission” (Zhou and Yi 2014). Parents are also often involved in interventions focusing on reducing anxiety in children and adolescents with ASD, often serving as “co-therapists”, encouraging the child to use strategies in anxiety-provoking situations and helping with homework completion (Sofronoff et al. 2005), and modeling courageous behaviors and coping strategies (Reaven 2010).

Parent co-regulation, defined as a parent’s support of their child’s emotional development through motivational or emotional scaffolding, and using strategies to help their child regulate emotions (Gulsrud et al. 2010), may be an instrumental set of processes to support the development of emotion regulation. As described by Hoffman et al. (2006), motivational scaffolding includes parents’ ability to initiate and sustain their child’s enthusiasm for a task, and may be shown through praise and encouragement, persistence, redirection of the child’s attention, or re-stating the goals of the task. Emotional scaffolding describes the parent’s ability to make the task a positive experience for the child, which is demonstrated by maintaining sensitivity towards the child’s emotions, sharing in the child’s positive emotions, and valuing the child’s participation in the task (Hoffman et al. 2006). Such emotional coaching is associated with lower child physiological stress and fewer externalizing problems in typically developing children and those with ASD (Hooven et al. 1995; Wilson et al. 2013). One advantage of exploring parent emotion co-regulation as a correlate of child emotion regulation and psychopathology is that it can be measured through behavioral observation (e.g., Lougheed et al. 2014).

Gulsrud and colleagues (2010) were the first to adapt a behavioral coding scheme to investigate co-regulation in mothers of toddlers with ASD (adapted from Grolnick et al. 1996), in a study of a 24-session joint attention intervention. Using this adapted coding scheme to code parent and child behavior during 10-min play periods at the end of each session, they found that co-regulation strategies used in mothers of typically developing toddlers (Grolnick et al. 1996) and those with ASD tended to be similar, though the ASD group tended to use more physical and active (e.g., physical comfort) ones. The authors also found that, using a global emotional and motivational scaffolding scale, mothers demonstrated improved co-regulation (i.e. higher ratings of global motivational and emotional scaffolding, higher frequency of more adaptive strategies such as redirection of attention) over the course of the intervention, and that this was also associated with improvements in toddler emotion regulation (i.e. less expressed negativity and avoidance).

### Current Study

Most of the existing literature on parent co-regulation and emotion regulation in children with ASD has focused on toddlers or young children (i.e., under 8 years of age), and because parents’ role in children’s emotional development is known to change as a child transitions from young childhood to adolescence (Reaven 2010), there is a need to investigate parent co-regulation in school-age children to determine possible changes over development. As well, few studies have used behavioral coding to measure parent co-regulation in ASD research. Most importantly, there is a need to investigate whether parent co-regulation and child ER might predict emotional and behavioral problems in youth with ASD.

To address these gaps, this study focused on co-regulation in parents of children with ASD between 8 and 12 years of age. We used a multi-method approach including behavioral observation, parent interviews, and open-ended measures, to address three questions. First, what types of co-regulation strategies do parents of school-age children with ASD use? Second, what are the associations amongst parent co-regulation, child emotion regulation, and child externalizing and internalizing problems? Lastly, do child emotion regulation skills and parent co-regulation predict psychopathology in school-age children with ASD over and above child characteristics (e.g., age and IQ)? We hypothesized that parents of school-age children with ASD would use passive and active co-regulation strategies more often than vocal strategies, similar to the transition from active to passive strategies observed in mothers of toddlers without ASD. We also hypothesized that more frequent use of co-regulation strategies, higher parent scaffolding, and child emotion regulation would be associated with lower levels of child externalizing and internalizing problems. Lastly, parent scaffolding and child ER were expected to emerge as significant predictors of child externalizing and internalizing problems, after accounting for child age and IQ.

## Method

### Participants

All participants were from the Greater Toronto Area and were enrolled in a randomized controlled trial of CBT to improve emotion regulation in children with ASD, 8 to 12 years of age (*M* = 9.65, *SD* 1.34). Data collection was based on the baseline data collection period including all children up until November 2015 (*N* = 51), prior to any treatment condition allocation. Of these 51 participants, three were excluded due to incomplete data. The following inclusion criteria was used: (a) a confirmed ASD diagnosis from available clinician reports or the *Autism Diagnostic Observation Schedule* (*ADOS*; Lord et al. 2008), as well as scores above the cut-off on the *Social Communication Questionnaire* (*SCQ*; Rutter et al. 2003) or the *Social Responsiveness Scale, Second Edition* (*SRS-2*; Constantino and Gruber 2012); (b) at least average intellectual functioning (IQ >79)^1^ on the two-subtest scale (FSIQ-2: vocabulary and matrix reasoning) of the *Wechsler Abbreviated Scale of Intelligence-2nd Edition* (*WASI-II*; Wechsler 2011); (c) between the ages of 8 and 12 years; and (d) demonstrated willingness to attend research assessments and 10 weekly therapy sessions. The majority of parents in this sample were mothers (77%, *N* = 37), and had a mean age of 44.09 years (*SD* 3.94). Further child characteristics are shown in Table 1. Children did not differ in any clinical or demographic characteristics depending on whether the participating parent was a mother or father (all *p* > .05).

**Table 1:** Child characteristics

	*M* (*SD*) or *N* (%)	Range
Age	9.65 (1.34)	8.00–12.00
Gender (% male)	42 (88%)	–
IQ	103.65 (13.84)	79.00–140.00
ASD symptomatology		
SCQ total score	21.00 (3.55)	14.00–27.00
SRS total T score	72.75 (9.23)	54.00–90.00

### Measures

#### Child Emotion Regulation

We used two open-ended measures to assess for emotion regulation ability that have been used previously with children with ASD (Beaumont et al. 2015; Beaumont and Sofronoff 2008; Sofronoff et al. 2005): *Dylan is Being Teased* (Attwood 2004a) and *James and the Maths Test* (Attwood 2004b). Both of these measures assess a child’s knowledge of appropriate emotion regulation strategies when given two hypothetical situations. Children’s verbal responses were written verbatim. Each appropriate strategy described was scored as one point (with no maximum limit), and the scores from the two measures were summed. Higher scores indicate a greater knowledge of appropriate strategies to use when experiencing anger or anxiety. The current sample had scores ranging from 0 to 14, with an average score of 4.13 (*SD* 3.43).

#### Parent Co-Regulation

To measure parent co-regulation strategies, we used a behavioral coding scheme previously used with mothers of typically developing children (Grolnick et al. 1996) and children with ASD (Gulsrud et al. 2010), and acceptable inter-rater reliability (*k* = 0.69 to 0.96 and *k* = 0.72 to 0.84, respectively). The coding scheme was used to code parent and child behaviors during a standardized *Emotion Discussion Task* (Suveg et al. 2008), in which each dyad was asked to discuss a time when the child felt anxious, angry, and happy (5 min per emotion). For the current study, co-regulation was coded on the two distressing emotions (anger and anxiety). This task has been used to assess parents’ roles in the emotional development of children with anxiety disorders (Suveg et al. 2008). Using 30-s partial-interval recording, we created composite scores for parent co-regulation strategies. The three parent co-regulation composites include: vocal (i.e., vocal comfort, reassurance), active (i.e., prompting/helping, redirection of attention, physical comfort), and following (i.e., following the child’s lead, emotion following). Our lab obtained good inter-rater reliability across two independent raters with this coding scheme (*k* = 0.67 to 0.97); 30% of the videos were double-coded for reliability. Table 2 lists definitions and examples of each co-regulation strategy.

**Table 2:** Parent co-regulation strategies definitions, adapted from Gulsrud et al. 2010

Behavior	Definition
Vocal composite	
Vocal comfort	Parent initiates vocalizations to comfort the child’s present emotional state (e.g., sshing, singing, sing-song voice, “It’s okay…”)
Reassurance	Parent reassures or encourages child surrounding frustrating or negative emotion discussion or emotions elicited by task (e.g., “It’s okay. You can do it!”, “It’s okay, we can talk about this together”, “It’s okay to feel…”, “I know it’s hard to talk about…”)
Active composite	
Prompting/helping	Parent physically or vocally prompts and scaffolds child or helps think of a time that he/she was feeling specific emotion (e.g., “Do you remember what you did/said next?”)
Redirection of attention	Parent directs the child’s attention to the discussion topic in an adaptive way (e.g., redirects conversation if child is perseverating on negative aspects of the discussion or goes off topic)
Physical comfort	Parent initiates behaviors to comfort the child’s present emotional state (e.g., hugs, kisses, offers a drink of water, rubs shoulder, touches hand)
Following composite	
Following the child’s lead	Parent is sensitive to child’s interests and responds to the child’s initiations in the conversation letting the child direct conversation and which event they choose to discuss (e.g., Child: “And then we went for pizza.” Mom: “That’s right, we did go for pizza…”)
Emotion following	Parent’s reflection, extension, or elaboration upon child’s past or present emotional state (e.g., “I know you were frustrated when that happened…”, “It seemed like you were feeling… when that happened.”, “You seem anxious right now...”)

For a measure of the quality of parent co-regulation, we also assigned global ratings for parent (a) motivational and (b) emotional scaffolding using a 5-point Likert scale (Gulsrud et al. 2010), ranging from 1 = “Parent exhibits characteristic ineffectiveness in scaffolding in a particular domain (e.g., emotional or motivational)—child’s needs for scaffolding are not met” to 5 = “Parent meets the child’s scaffolding needs almost the entire time; there may be a rare instance in which the parent misses a minor opportunity for scaffolding.” Motivational scaffolding refers to parents’ ability to help the child maintain enthusiasm toward the task, including praise and encouragement, and redirecting attention back to the conversation topics. Emotional scaffolding is parents’ ability to make the task a positive experience for the child, which includes valuing the child’s participation in the task and maintaining sensitivity towards the child’s emotions. Inter-rater reliability for these global scaffolding scores was good (*k* = 0.67). Mean motivational scaffolding was correlated with mean emotional scaffolding across all conditions, *r* (46) = 0.72, *p* < .001, and for each emotion condition (*r*’s ranged from 0.58 to 0.64). As a result, the mean of the motivational and emotional scaffolding scores was calculated for each emotion condition.

#### Child Psychopathology

Externalizing and internalizing problems were measured via parent report on the Externalizing and Internalizing subscales of the *Behavior Assessment System for Children, Second Edition*—*Parent Rating Scales* (*BASC-2*; Reynolds and Kamphaus 2004), used previously to study emotional and behavioral problems in youth with ASD (Volker et al. 2010), and found to have high internal consistency (*α* = 0.81–0.94), test re-test reliability (*r* = .88–.91), and moderate to high concurrent validity (*r* = .53 to .83; Reynolds and Kamphaus 2004). For the Internalizing subscale, 15 participants in the current sample (31%) scored in the At-Risk range, and 11 (22%) scored in the Clinically Significant range. For the Externalizing subscale, 10 participants (21%) scored in the At-Risk range, and 6 (12%) scored in the Clinically Significant range.

### Procedures

The university Research Ethics Board approved data collection for this study and all parents provided informed consent and children, assent. Participants were recruited through local autism service e-newsletters, website postings, and referrals from doctors in the community. Participants first completed a telephone screening with a research assistant to confirm that their child has an ASD diagnosis and was between 8 and 12 years of age, and participants completed the *SCQ* (Rutter et al. 2003) and *SRS-2* (Constantino and Gruber 2012). Participants then took part in an in-person screening, where researchers administered the *WASI-II* (Wechsler 2011) with the child and evaluated the family’s willingness to attend research assessments and therapy sessions. After this screening process, children completed the James/Dylan task, parents completed the BASC-2, and the dyads participated in the Emotion Discussion Task, which formed the basis for the behavior co-regulation coding. Families were reimbursed for travel expenses, and each child was given a small prize (e.g. notebook, ball) at the end of each research testing appointment. All participants who met inclusion were included in the current study, regardless of their progress or involvement in subsequent treatment; the following data analyses are based on the baseline data collection.

### Data Analysis

All statistical analyses were performed using IBM SPSS version 21. Pearson product-moment correlations were calculated to examine the relationships among all predictor variables and child externalizing and internalizing problems. A multiple regression analysis was conducted for predictor variables with significant correlations with child externalizing and internalizing problems. To avoid violating normal distribution assumptions given the limited sample size, 1000 bootstrap samples were drawn as an estimation of direct effects (Farmer 2012; Preacher and Hayes 2008). Child age and IQ were entered as covariates in all analyses.

## Results

As expected by our first hypothesis, Active and Following co-regulation strategies were more commonly used than Vocal strategies. As shown in Table 3, Following strategies were more frequently observed than Active strategies [*t*(47) = 5.94, *p* < .001], and Active strategies were more frequently observed than Vocal strategies [*t*(47) = 21.47, *p* < .001]. The two most commonly observed co-regulation strategies were one Active form (prompting/helping; *M* = 22.83, *SD* 4.78), and one Following form (emotion following; *M* = 21.90, *SD* 5.10). Vocal comfort (*M* = 0.08, *SD* 0.40) and reassurance were least observed (*M* = 1.73, *SD* 1.67), and in fact, vocal comfort was only observed in one parent. Mothers demonstrated an equal number of overall Active, Following, and Vocal strategies compared to fathers, nor did they differ in any form of scaffolding (all *p* > .05). With regard to specific co-regulatory behaviors, mothers showed similar levels as fathers in all but one area: mothers demonstrated significantly more physical comfort (*U* = 122.5, *p* = .02), an Active strategy, compared to fathers.

**Table 3:** Descriptive statistics for parent co-regulation strategies

Variables	*Mean*	*Median*	*SD*	*Range*
Vocal composite	0.91	0.50	0.91	0.00–3.50
Vocal comfort	0.08	0.00	0.40	0.00–2.00
Reassurance	1.73	1.00	1.67	0.00–6.00
Active composite	9.47	9.50	2.72	3.67–18.00
Prompting/helping	22.83	23.00	4.78	9.00–30.00
Redirection of attention	3.65	2.00	3.52	0.00–15.00
Physical comfort	1.92	0.00	3.15	0.00–15.00
Following composite	12.58	12.50	2.78	4.00–20.50
Following the child’s lead	3.27	3.00	3.79	0.00–20.00
Emotion following	21.90	23.00	5.10	5.00–30.00

Pearson product-moment correlations were conducted to address the second research question, investigating the associations among all predictor variables and child externalizing and internalizing problems. As shown in Table 4, child internalizing problems had a marginally significant association with child emotion regulation [*r*(46) = −0.26, *p* = .08], and significant associations with child age and ASD severity. Child externalizing problems were significantly associated with parent scaffolding in both the angry [*r*(46) = −0.32, *p* = .03] and anxious conditions [*r*(46) = −0.34, *p* = .02], as well as with child emotion regulation ability [*r*(46) = −0.30, *p* = .04]. None of the specific co-regulation strategies were significantly related to internalizing or externalizing problems, although physical comfort was marginally significant with internalizing problems [*r*(46) = −0.25, *p* = .08].

**Table 4:** Correlations among potential predictor and dependent variables

	Predictor variables								Dependent variables	
	2	3	4	5	6	7	8	9	Int. prob.	Ext. prob.
1. Child age	−.19	.09	−.33*	−.36*	.02	−.49***	.04	−.02	.29*	.06
2. Child IQ		−.24	.06	.02	.15	.06	.05	−.06	−.22	−.30*
3. ASD severity			−.03	−.05	−.07	.11	.33*	−.14	.41**	.24
4. Scaffolding: anxious				.87***	.17	.43**	.36*	.11	−.18	−.34*
5. Scaffolding: angry					.11	.29*	.17	.07	−.14	−.33*
6. Co-regulation: vocal						.12	−.15	−.09	−.02	−.01
7. Co-regulation: active							.12	.07	−.13	−.02
8. Co-regulation: following								.10	−.08	−.19
9. Child ER									−.26^+^	−.31*

^+^ *p* < .10, *p < .05, **p < .01, ***p < .001

Given the strong correlation between scaffolding in the angry and anxious conditions, a mean scaffolding score was calculated across conditions for the regression analyses involving externalizing problems as the dependent variable. The mean scaffolding score was correlated with externalizing problems [*r*(46) = −0.33, *p* = .02], but not internalizing problems [*r*(46) = −0.16, *p* = .27]. Due to the non-significant associations between child internalizing problems and the parent co-regulation variables, the following hierarchical regression analysis focused on predictors of child externalizing problems. Table 5 displays the hierarchical regression model for the predictors of child externalizing problems. The overall model accounted for 29% of the variance in externalizing problems, with significant changes in variance at Step 2, suggesting that parent scaffolding and child emotion regulation had an effect above and beyond the effects of child age and IQ [*ΔR* ^*2*^ = 0.20, *F*(4, 41) = 4.09, *p* = .007], as expected by our third hypothesis. Looking at these variables individually, child IQ (*p* = .02), parent scaffolding (*p* = .03), and child emotion regulation (*p* = .03) emerged as significant predictors.

**Table 5:** Parent scaffolding and child emotion regulation as predictors of child externalizing problems

Variable	*B*	*SE*	*t*	*R*	*R* ^*2*^	*ΔR* ^*2*^
Step 1				.30	.09	.09
Constant	76.67***	15.06	5.03			
Child age	.23	.98	.24			
Child IQ	−.19^+^	.10	−1.95			
Step 2				.53	.29	.20
Constant	100.74***	16.48	6.11			
Child age	−.56	.95	−.59			
Child IQ	−.21*	.09	−2.37			
Parent scaffolding	−3.25*	1.47	−2.21			
Child ER	−.79*	.35	−2.26			

^+^ *p* < .10, *p < .05, **p < .01, ***p < .001

## Discussion

This is the first study to use observational methods to investigate parent co-regulation and emotion regulation in school-age children with ASD, building on what is known in the context of toddlers with ASD (Gulsrud et al. 2010). Focusing on co-regulation composite scores, parents of school-age children with ASD used significantly more passive co-regulation strategies (i.e., following) than active ones. However, upon closer examination of the specific co-regulation strategies used, prompting (an active strategy) and emotion following (a passive strategy) were commonly observed. Prompting involves parental guidance and structuring of a child’s emotional experience by taking the lead of the discussion, helping the child to think of an event to discuss and asking the child to elaborate on aspects of an event. Emotion following, in contrast, allows the child to direct the emotional discussion.

The balance between the structure provided by prompting and the child-led opportunities provided by following may strike the right balance to fit with the current needs of school-age children with ASD who have at least average intellectual functioning. Gulsrud and colleagues (2010) noted that mothers of toddlers with ASD used primarily physical and active co-regulation strategies, rather than the more passive, verbal strategies found in mothers of toddlers without ASD, as a result of maternal sensitivity to children’s developmental needs. The combination of active and passive co-regulation strategies with school-age children with ASD may be most beneficial as prompting helps guide a child’s emotional experience, while emotion following helps a child internalize adaptive emotion regulation skills (Cole et al. 2009). As Hoffman (1983) argues, providing both structure and freedom can create a context that is “somewhat but not overly arousing”, and thus best for instilling emotional growth. This may be of particular importance when parenting a child with ASD, given that some studies have found that children with ASD have higher baseline levels of arousal (i.e., higher heart rate) and a blunted heart rate response during a social stressor (Jansen et al. 2003, 2006). Being able to assist children to lower their levels of physiological arousal may be an important way of helping cope with environmental stimuli.

Our sample also demonstrated very few vocal strategies (i.e., vocal comfort, reassurance), likely because these strategies are more developmentally appropriate for toddlers than school-age children, and more appropriate during expressions of child negativity (Gulsrud et al. 2010). Most children (67%, *n* = 32) in the current study did not display any physical or verbal venting or tension release (e.g., kicking, yelling); behaviors that may elicit vocal comfort or reassurance from parents, and that were observed far more often in preschool age children (Gulsrud et al. 2010). Gulsrud and colleagues (2010) observed more parent vocal comfort and reassurance when children displayed physical or verbal negativity than when children did not display negativity. It is also possible that children with more severe ASD symptomatology or lower IQ, who were not included in the current study, would require even more active co-regulation strategies than reported here, because of children’s lesser ability to take lead of the emotional discussion and their greater need for prompting and guidance from their parents.

The current study found that parent scaffolding was associated with child externalizing problems. Parent scaffolding, which taps into parents’ ability to respond sensitively to their child and maintain their child’s persistence toward the task, is important in children’s emotional development. Wilson and colleagues (2013) found that higher levels of parent emotion coaching (a concept similar to parent scaffolding) were associated with lower levels of externalizing problems in children with ASD. The authors found that this association was stronger in children with ASD than in typically developing children, possibly because those with ASD generally had more externalizing problems and required more support from their parents. Motivational and emotional scaffolding may be important because of its relation to positive parenting (e.g., showing warmth, positivity, and acceptance) (Eisenberg et al. 1998; McCarty et al. 2005). Through the perspective of positive parenting, parents who display responsiveness and child-centered caring promote healthy emotion regulation, and in turn, have children who are less likely to exhibit externalizing problems (McCarty et al. 2005; Wilson et al. 2013). Our findings may also lend support to some emerging research on the efficacy of Parent Child Interaction Therapy (PCIT) with parents of children with ASD and externalizing behavior problems (Masse et al. 2016).

Contrary to our hypothesis, the frequency of parent co-regulation strategies was not associated with child externalizing problems. The behavioral coding of co-regulation was a simple count of the number of 30-s intervals where the specific strategy was observed, which is qualitatively distinct from the ratings of parents’ motivational and emotional scaffolding that takes into account how effective parents help their child and the degree to which their child’s needs for scaffolding were met. The frequencies of co-regulatory behaviors may be less important than the quality of their overall use.

Our results support past findings that children with better emotion regulation ability tend to have lower parent-reported externalizing problems (e.g., Mazefsky et al. 2014; Rieffe et al. 2011). The current study extends what is known by being the first to demonstrate that children’s knowledge of emotion regulation strategies, as coded through child report, are also related to parent reports of externalizing problems. Given the high rates of emotional difficulties (Ooi et al. 2011; Totsika et al. 2011), psychopathology (Brereton et al. 2006; Dickerson et al. 2011), and externalizing and internalizing problems (Maskey et al. 2013) in children with ASD, these findings support the need for interventions targeting the underlying deficits in emotion regulation abilities (Gross and Thompson 2007; Mazefsky et al. 2013; Rieffe et al. 2011; Weiss 2014). The majority of CBT interventions for children with ASD have focused on anxiety (e.g., Reaven et al. 2012), but an emotion regulation framework may allow interventions to address both internalizing and externalizing problems in this population (Weiss 2014). Even after taking into account the relative contribution of age and IQ, parents’ scaffolding, and children’s ability to regulate their emotions, are important factors in understanding externalizing problems. They may also serve as helpful areas of focus in interventions targeting children’s externalizing symptoms.

It is interesting to note that internalizing problems were not associated with parent scaffolding nor co-regulation strategies, and only marginally associated with children’s emotion regulation abilities. This may be because the quality of parent emotional support is less relevant to child internalizing problems than the child’s own knowledge of appropriate emotion regulation strategies. Previous studies have found a similar pattern of results, with parent scaffolding and positive parenting being more strongly associated with child externalizing problems than internalizing problems (Boonen et al. 2014; Hoffman et al. 2006; McCarty et al. 2005). Considering that depressive disorders typically do not emerge until adolescence, McCarty and colleagues (2005) suggest that the effect of parent scaffolding and emotional support on child internalizing problems may not be seen until later in development.

### Limitations and Future Research Directions

Generalizability of these findings may be limited in that the sample consisted of parents who were seeking treatment for their child’s emotional problems. These parents may exhibit different co-regulation strategies than parents who are not seeking treatment. In addition, all children had an IQ above 79, and it is unclear how the current findings might differ for school-age children with more severe ASD symptomatology or lower intellectual functioning. This study also could have benefited from using multiple measures of child emotion regulation (e.g., parent report, behavioral observation, psychophysical measurement; Weiss et al. 2014) instead of relying solely on coding child report of emotion regulation strategies.

Due to the transactional nature of the relationship between child self-regulation and parent co-regulation (Sameroff and Fiese 2000), as well as this study’s reliance on correlational data, it is difficult to determine directionality between parent and child regulation. Future longitudinal research in this area is required, and pre-post intervention data could examine parent co-regulation as a mechanism to explain treatment efficacy in children with ASD. Further research could also investigate the types of co-regulation strategies used by parents of typically developing school-age children. This study adapted a behavioral coding scheme that was originally used with younger children with ASD and their mothers (Gulsrud et al. 2010). As a result, our results may not fully capture the more complex verbal strategies employed by parents of school-age children. However, without a comparison group, it is difficult to determine whether parents of children with ASD use different co-regulation strategies than do parents of typically developing children.

## Conclusion

Using a multi-method approach, this study demonstrated that children’s knowledge of appropriate emotion regulation strategies, and the quality of parent scaffolding in distressing situations, are associated with parent-reported psychopathology in children with ASD. Specifically, parental active and passive co-regulation and overall scaffolding have important relations to child externalizing problems, and interventions for children with ASD targeting emotion regulation should encourage parents to use scaffolding techniques when their child is exhibiting anger or overly emotional arousal. Overall, parent–child interactions are important in understanding child mental health, and parents continue to play a fundamental role in their children’s emotional development, beyond toddlerhood and into school-age years. With future research in the topic, parent co-regulation and scaffolding may emerge as useful areas of focus in interventions targeting psychopathology in children with ASD.

## Footnotes

1 One child with an overall IQ of 79 was included as a result of average scores on the Vocabulary subtest of the *WASI-II*.
